# Inhibition of MLC Phosphorylation Restricts Replication of Influenza Virus—A Mechanism of Action for Anti-Influenza Agents

**DOI:** 10.1371/journal.pone.0021444

**Published:** 2011-06-23

**Authors:** Mehran Haidari, Wei Zhang, Leila Ganjehei, Muzammil Ali, Zhenping Chen

**Affiliations:** 1 Department of Internal Medicine, Division of Cardiology, University of Texas Health Science Center at Houston, Houston, Texas, United States of America; 2 Texas Heart Institute at St. Luke's Episcopal Hospital, Houston, Texas, United States of America; University of Delhi, India

## Abstract

Influenza A viruses are a severe threat worldwide, causing large epidemics that kill thousands every year. Prevention of influenza infection is complicated by continuous viral antigenic changes. Newer anti-influenza agents include MEK/ERK and protein kinase C inhibitors; however, the downstream effectors of these pathways have not been determined. In this study, we identified a common mechanism for the inhibitory effects of a significant group of anti-influenza agents. Our studies showed that influenza infection activates a series of signaling pathways that converge to induce myosin light chain (MLC) phosphorylation and remodeling of the actin cytoskeleton. Inhibiting MLC phosphorylation by blocking RhoA/Rho kinase, phospholipase C/protein kinase C, and HRas/Raf/MEK/ERK pathways with the use of genetic or chemical manipulation leads to the inhibition of influenza proliferation. In contrast, the induction of MLC phosphorylation enhances influenza proliferation, as does activation of the HRas/Raf/MEK/ERK signaling pathway. This effect is attenuated by inhibiting MLC phosphorylation. Additionally, in intracellular trafficking studies, we found that the nuclear export of influenza ribonucleoprotein depends on MLC phosphorylation. Our studies provide evidence that modulation of MLC phosphorylation is an underlying mechanism for the inhibitory effects of many anti-influenza compounds.

## Introduction

**Figure 1:**
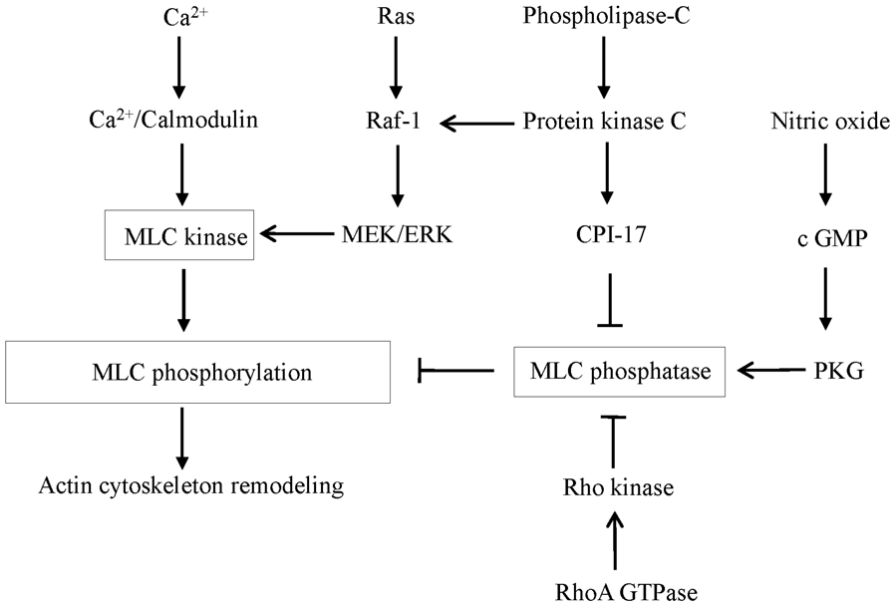
Pathway model used in this study. Signal transduction pathways involved in myosin light chain phosphorylation. MLC, myosin light chain; PKG, protein kinase G.

The emergence of highly contagious influenza A virus strains, such as the new H1N1 swine influenza, is a serious threat to global human health. Two classes of anti-influenza agents are currently available for use during an influenza pandemic-M2 channel blockers and neurominidase inhibitors. However, continuous antigenic changes enhance the probability of increased resistance to these drugs. Moreover, a high frequency of resistance in clinical isolates in the United States has led to the conclusion that M2 inhibitors should not be used for the prevention or treatment of influenza until susceptibility to these drugs has been re-established among circulating influenza A isolates [1]. Because of their relatively small genomic coding capacity, influenza A viruses extensively manipulate and exploit host cell functions to support viral replication. Therefore, targeting cellular proteins required for influenza replication is a valuable alternative for preventing and treating infections. This approach is advantageous in that the development of drug resistance is unlikely and the drugs target common pathways used by human, avian, and other influenza viruses. However, this strategy requires an understanding of the intracellular pathways that the influenza virus uses to replicate. The actin cytoskeleton plays a critical role in viral replication [2]. Contraction and relaxation of the actin cytoskeleton are primarily regulated by phosphorylation and dephosphorylation of the regulatory subunit of myosin light chain (MLC) [3]. Phosphorylation of MLC is controlled by a balance of activation and deactivation of myosin light chain kinase (MLCK) and myosin light chain phosphatase (MLCP) [4]. The classic pathway through which contracting stimuli induce MLC phosphorylation is by coupling their receptors to heterotrimeric G proteins, resulting in the activation of phospholipase C (PLC) beta isoforms, the formation of inositol-1, 4, 5-trisphosphate, and an increased concentration of free cytosolic Ca^+2^. The complex of Ca^+2^ and calmodulin then activates MLCK, leading to increased MLC phosphorylation (Fig. 1). The Ca^+2^-independent regulation of actin-myosin contraction occurs through the inhibition of MLCP and involves other biochemical cascades, including the monomeric GTP-binding protein RhoA, protein kinase C (PKC), and the Ras/Raf/MEK/ERK signaling cascade [4], [5], [6]. Activation of RhoA leads to the stimulation of Rho-kinase, which, in turn, phosphorylates the regulatory myosin-binding subunit of MLCP, resulting in the inhibition of MLCP. Another pathway for inhibiting MLCP involves the activation of PKC, which leads to phosphorylation and activation of CPI-17. Furthermore, activation of the HRas/Raf/MEK/ERK signaling cascade also leads to activation of MLCK and inactivation of MLCP [6], [7], [8]. Nitric oxide (NO) increases cGMP concentration by activating guanylyl cyclase, which, in turn, activates protein kinase G (PKG). PKG inhibits MLC phosphorylation by reducing intracellular Ca^+2^ levels and activating MLCP [3], [9].

Influenza-induced activation of Raf/MEK/ERK, PKC, and PLC has been reported [10], [11], [12]. Influenza infection also leads to an increase in intracellular calcium levels and actin polymerization [13]. These studies suggest that the signal transductions that are involved in MLC phosphorylation and actin cytoskeleton remodeling are activated after influenza infection. Furthermore, it is well documented that the inhibition of Raf/MEK/ERK and PKC signaling pathways leads to inhibition of influenza proliferation [12], [14]. In addition to MEK/ERK and PKC inhibitors, calcium channel blockers, calmodulin inhibitors, NO donors, and agents that restrict actin polymerization have anti-influenza effects [15], [16], [17], [18]. However, the underlying molecular mechanisms for these anti-influenza properties have not been determined. The common thread among these agents is the inhibition of MLC phosphorylation and alteration in actin contractile function (Fig. 1). Thus, in the present study, we tested the hypothesis that inhibition of MLC phosphorylation leads to inhibition influenza virus replication.

## Materials and Methods

### Reagents

Phospho-specific and nonphospho-specific antibodies against ERK1/2, nonmuscle myosin II, and fluorescein isothiocyanate (FITC)-conjugated goat anti-mouse secondary and anti-FITC–HRP antibodies were obtained from Abcam. Monoclonal phospho-antibody against MLC and specific antibody against MLC were purchased from Sigma-Aldrich. Phospho-specific and nonphospho-specific antibodies against MLCK and the catalytic subunit of MLCP (MYPT) were obtained from Santa Cruz Biotechnology. Recombinant TNF-α, PKC activator (Phorbol-12-myristate-13-acetate, PMA), RhoA inhibitor (C3 exoenzyme), Rho kinase inhibitors (Y-27632, Fasudil), MLCK inhibitor (ML-7), MEK inhibitors (U0126, PD-98059), protein kinase C inhibitor (bisindolylmaleimide I), phospholipase C inhibitor (U73122), Raf-1 kinase inhibitor (GW 5074), calmodulin antagonist (trifluoperazine dimaleate), intracellular Ca^2+^ chelator (BAPTA-AM), calcium channel blocker (verapamil), MLCP inhibitor (calyculin A), sodium nitroprosside, 8-Br-cGMP, and cytochalasin D were purchased from Calbiochem (San Diego, CA). LFL was purchased from EY Laboratories, Inc. Tyramide signal amplification kits (TSA, Fluorescein) were obtained from PerkinElmer. Anti-influenza monoclonal antibody against nucleoprotein was purchased from Chemicon International, Inc. Premade recombinant adenoviruses of ERK2 (DN), Ras V12 (CA), Ras N17 (DN), Raf-1 (DN), RhoA N19 (DN), RhoA L63 (CA), null control, GFP, and ViraDuctin adenovirus transduction reagents were purchased from Cell Biolabs. A mutant construct for DN MLCK was a generous gift from Dr. Yasuo Mori (National Institute for Physiological Sciences, Okazaki, Aichi, Japan). Small interfering RNA (siRNA) and TaqMan primers and probes for MYPT, Rho kinase1/2, Gα12/13, and Gαq11 were purchased from Applied Biosystems.

### Cells

HUVECs and MDCK cells were purchased from American Type Culture Collection. NHBE cells were purchased from Lonza (Rockland, ME). MDCK cells were grown in Dulbelcco's Modified Eagle Medium (DMEM)+10% FBS. HUVECs and NHBE were grown in Endothelial Cell Growth Medium-2 (EGM-2, Lonza) and Bronchial Epithelil Cell Growth Medium (BEGM, Lonza), respectively. HUVECs and NHBE cells from fewer than 4 generations were used for all experiments.

### Cell viability assay

The effects of chemicals on the viability of MDCK cells and HUVECs were determined by using the 3-[4, 5-dimethylthiazol-2-yl]-2, 5-diphenyl tetrazoliumbromide (MTT) assay, as previously described [19].

### Viral infection

Influenza virus A/Hong Kong/2/68; H3N2 [A/HK (H3N2)] was used as the primary influenza virus strain in all experiments. When grown to 90% confluency, HUVECs or MDCK cells were washed twice with PBS to remove residual fetal bovine serum (FBS) and were infected with virus at an MOI of 10 or 0.01 to allow single-cycle (8–10 h after infection) or multicycle (24 h after infection) replication, respectively. Viral stock was used in serum-free DMEM for 60 minutes at 37°C to inoculate the cells (adsorption phase). Cells were then washed with PBS and cultured in DMEM+2% FBS and tolylsulfonyl phenylalanyl chloromethyl ketone (TPCK) trypsin (4 µg/ml, Worthington Biochemicals, Tryp-MEM ), either with or without drug treatment, for 8 to 24 h (postinfection phase).

### Extracellular virus yield reduction assay

These assays were performed in 24-well plates containing confluent MDCK monolayers (4 times for each experiment). The drugs were added to cells in 24-well plates, and the plates were incubated for 0.5 to 8 h at 37°C. The cells were then inoculated with virus, first for 1 h, and then again after washing for 24 h at 37°C in DMEM+2% FBS and TPCK trypsin. The medium was removed and centrifuged at 3,200× g for 5 min to remove the floating cells and then used for ribonucleic acid extraction and quantification of virus by using qPCR.

### qPCR assay

TaqMan real-time PCR was used to quantify the presence of virus after infection with influenza virus, as described [19]. The agreement between conventional techniques used for influenza replication (eg, plaque assay and TCID 50%) with qPCR technique has been established [19]. We used qPCR because it was faster than conventional techniques (8 h vs 3–4 days). The primer and probe sequences for detection of influenza A were previously optimized [20]. The primers (forward) 5′ AAG ACC AAT CCT GTC ACC TCT GA 3′ and (reverse) 5′CAA AGC GTC TAC GCT GCA GTC C 3′ amplify a 104-base pair fragment in the *M1* gene of influenza A. The influenza A–specific probe FAM (6-carboxyfluorescein)-5′ TTT GTG TTC ACG CTC ACC GT 3′ TAMRA (6-carboxytetramethylrhodamine) annealed to part of the sequence amplified by the 2 primers.

### Immunofluorescence staining of influenza virus

Cells were fixed in 4% paraformaldehyde for 15 min at 4°C, washed with PBS, and permeabilized for 5 min with 0.1% Triton-X-100. Viral nucleoprotein (NP) protein was detected by using mouse monoclonal antibody (Chemicon International, Inc.). The secondary antibody used was FITC-conjugated goat anti-mouse IgG. Tyramide signal amplification system was used in conjunction with anti-influenza antibody. After quenching the remaining horseradish peroxidase (HRP) activity with 1% H_2_O_2_, 1 µg/ml anti-FITC–HRP (for 30 min) and FITC-Tyramide (Molecular Probes) were used to reveal FITC–anti-influenza staining. Hoechst 33342 (0.5 µg/ml for 5 minutes) was used as a nuclear counter-stain. Fluorescent images were acquired by MicroSuite FIVE software (Olympus Soft Imaging Solutions) with an Olympus BX61 motorized microscope (Olympus America).

### Western blot assay

Cell lysates were separated on sodium dodecyl sulfate (SDS)-polyacrylamide gels, transferred onto nitrocellulose membranes, and stained with primary antibodies, as indicated in figure legends. Secondary antibodies were conjugated to HRP (Sigma). Membranes were developed by use of an enhanced chemiluminescence system (Cell Signaling Technology, Inc.).

### Transduction of adenovirus and transfection of siRNA and plasmids

All reagents and kits, including transduction reagents, adenovirus purification kit, and adenovirus titration kit were purchased from Cell Biolabs, Inc. After purification, the titration of each recombinant adenovirus was determined by an enzyme-linked immunosorbent assay (ELISA) titrating kit. Null control recombinant adenovirus (empty vector) served as a control for other recombinant adenovirus used. HUVECs, NHBE or MDCK cells were seeded into 6-well plates for 24 h until they reached 80% confluency. According to the manufacturer's protocol, adenovirus was transduced into cells by using ViraDuctin (Cell Biolabs, Inc.). HUVECs or MDCK cells were infected with adenoviral vectors with an MOI of 100 plaque-forming units per cell in the presence of ViraDuctin. After incubation with viral particles for 48 h, the cells were assessed for the expression of the transduced genes. Plasmids and siRNA were transfected into cells by using Lipofectamine 2000 (Invitrogen). Scrambled siRNA (a non-targeting siRNA pool) or pcDNA3.1 empty vector were transfected as the controls. Cells were collected 24 h after transfection with siRNA or plasmids.

### RhoA, Rho kinase, and PKC activity assays

RhoA activity was determined by using a RhoA G-LISA Activation Assay kit according to the manufacturer's protocol (Cytoskeleton, Inc.). This assay is based on the principle that a Rho-GTP-binding protein is linked to the 96-well plates. The active GTP-bound Rho in the cell lysates binds to the wells, while the inactive GDP-bound Rho is removed during the washing steps. The bound active RhoA is detected with a RhoA specific antibody and quantified by absorbance. The degree of RhoA activation is determined by comparing readings from the infected cell lysates versus the non-infected cell lysates [21]. A commercially available kit was used to measure Rho kinase activity according to the manufacturer's instruction (CycLex Co., Ltd.; MBL International Corporation). In brief, the supernatants of lysed cells were measured for protein concentrations and justified to the same protein concentration and then added (10 µl/well) into 96-well plates precoated with a substrate corresponding to the C terminus of the recombinant myosin-binding subunit of myosin phosphate (MSB), which contains a threonine residue that may be phosphorylated by Rho kinase. Subsequently, 90 µl of kinase reaction buffer was added to the kinase reaction, incubated for 60 min at room temperature, washed five times in washing buffer provided in the kit, and incubated with 100 µl of horseradish peroxidase-conjugated monoclonal antiphospho-specific MSB antibody. The colored products were developed by incubating with 100 µl of the horseradish peroxidase substrate tetramethylbenzidine at room temperature for 10 min. The colored products were quantified by spectrophotometry at 450 nm [22]. Total PKC activity in cells was measured by using the PKC Assay kit from Calbiochem (San Diego, CA). The protocol established by the company was used as described [23]. A total of 108 µl of reaction was placed in each well of a polyvinyl plate and preincubated at 25°C for 5 min. Samples (12 µl), including PKC standards (the active PKC protein) were added to each well and mixed. Each sample was run in duplicate. A total of 100 µl of each sample in reaction mixture was transferred to pseudosubstrate- coated wells with a multichannel pipetter. After incubation at 25°C for 15 min, the reaction mixture was removed from the plate, and the plate was washed five times with PBS. Biotinylated Ab 2B9 (100 µl) directed to the phosphorylated pseudosubstrate was added to each well and incubated at 25°C for 60 min. Peroxidase-conjugated streptavidin (100 µl) was then added to each well and incubated for another 60 min. The wash was repeated after incubation and 100 µl of substrate solution (*o*-phenylenediamine) was added to each well. Stop solution (100 µl) was added after 3–5 min and the 96-well plate was read at 492 nm in a microplate reader.

### Measurement of intracellular calcium

Cells were loaded with 4 µg of fluo-3 acetoxymethyl (AM) and 10 µg of Fura-Red AM (Molecular Probes, Inc.) as described [24]. Flow cytometry analysis was performed at 488 nm excitation, and the fluorescence of fluo-3 was collected at 520 nm, and Fura-Red emission was collected at 640 nm. An in situ calibration assay for calcium measurements in HUVECs was performed as described [25]. The fluo-3/Fura-Red ratio versus calcium concentration was plotted and used to calculate [Ca^2+^]i in each sample as described [25].

### Actin assays

Actin polymerization was measured by quantifying the ratio of F actin to G actin by using a G actin/F actin assay kit (BK037; Cytoskeleton, Inc.). Actin fibers were stained by using rhodamine phalloidin from the F-actin visualization Biochem Kit (BK005; Cytoskeleton, Inc).

## Results

### Influenza A virus infects and replicates in HUVECs

Although the Madin Darby canine kidney (MDCK) cell line is most commonly used for influenza studies, we and others have shown that the influenza virus infects and replicates in human umbilical vein endothelial cells (HUVECs) [26], [27]. However, influenza replicates to a lesser extent in HUVECs than in MDCK cells [27]. Because the MDCK cell line is not a human cell line, antibody reagents and siRNAs are not readily available. Thus, we used MDCK cells mainly for studying the effects of inhibitors or stimuli on influenza replication, and we used HUVECs for signal transduction and siRNA experiments. As shown below the results obtained from HUVECs and MDCK cells were similar for both influenza-induced signal transductions and influenza intracellular trafficking and proliferation.

### Optimization of adenovirus transduction and siRNA and plasmid transfection

**Figure 2:**
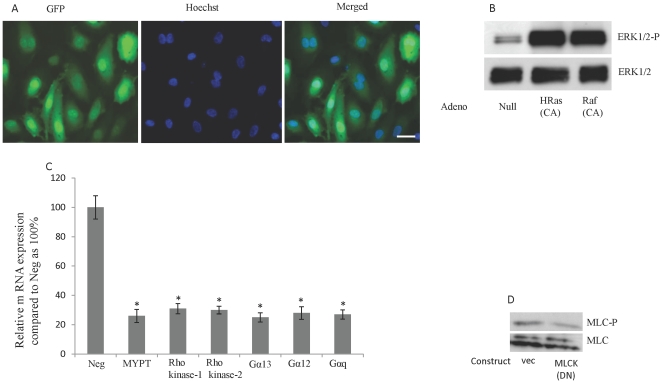
Validation of adenovirus transduction and siRNA and plasmid transfection. (A) The average transduction rate of HUVECs with the recombinant GFP-adenovirus was 80% to 90%. HUVECs were transduced with GFP adenovirus and after 48 h were assessed for transfection rate. Hoechst 33342 (0.5 µg/ml for 5 minutes) was used as a nuclear counter-stain. Fluorescent images were acquired by MicroSuite FIVE software (Olympus Soft Imaging Solutions) with an Olympus BX61 motorized microscope. Bar, 10 µm. (B) Transdution of HUVEC with CA-HRas and CA-Raf adenoviruses induced ERK phosphorylation. HUVECs were transduced with the indicated adenoviruses and 48 h were used for blotting. (C) Treatment of HUVECs with siRNA for MYPT, Rho kinase1/2, Gα12/13, and Gαq11 led to reduction in the mRNA expression. HUVECs were transfected with the indicated si RNA and after 24 h the m RNA leves for each gene were quatified by TaqMan real-time PCR . *P<0.01; N = 4 for each experiment. (D) Overexpression of the DN mutant of MLC kinase in HUVECs led to a 2.5-fold reduction in MLC phosphorylation. HUVECs were transfected with DN mutant of MLC kinase and 24 h later were used for blotting.

The transduction conditions for the adenovirus recombinants were optimized by using adenovirus-encoding green fluorescent protein (GFP). The average transduction rate of HUVECs and MDCK cells with the recombinant GFP-adenovirus was 80% to 90% (Fig. 2A). To confirm the efficiency of transductions for the adenovirus recombinants that were used in the study, we tested either the function of the proteins (eg, pull-down assay for HRas and RhoA and ELISA activity assay for PKC) or the downstream effectors of the genes. Transduction of HUVECs with HRas and Raf led to increase in ERK phosphorylation (Fig. 2B). A FITC-labeled, double-stranded siRNA (Invitrogen) was used to optimize the transfection of siRNA into HUVECs and normal human bronchial epithelial (NHBE). The siRNA for MYPT, Rho kinase1/2, Gα12/13, and Gαq11 were validated by Applied Biosystems. To confirm the efficiency of siRNA transfection, we used TaqMan real-time PCR to measure the mRNA expression of genes of interest. The average siRNA transfection rate was 70%, and we observed an average 3–4–fold decrease in the mRNA expression of MYPT, Rho kinase1/2, Gα12/13, and Gαq11 in cells treated with the respective siRNA (Fig. 2C). The vector pcDNA3.1/CT-GFP TOPO (Invitrogen) was used to optimize the transfection of MLCK plasmids into HUVECs and MDCK cells. The average transfection rate for pcDNA3.1/CT-GFP was 30% to 40%. Inhibition of MLC kinase in HUVECs by overexpression of the DN mutant of MLC kinase led to a 2.5-fold reduction in MLC phosphorylation (Fig. 2D).

### Influenza infection induces phosphorylation of MLC

Infection of permissive cells with influenza viruses induces activation of several intracellular signaling pathways that are then used by the virus for its own replication [28], [29]. Specifically, influenza infection induces activation of the Raf/MEK/ERK signaling cascade, which is required for virus replication [12]. The mechanism by which ERK activation facilitates influenza replication is unknown; however, direct phosphorylation of a viral protein by MEK and ERK is not believed to be involved [12].

**Figure 3:**
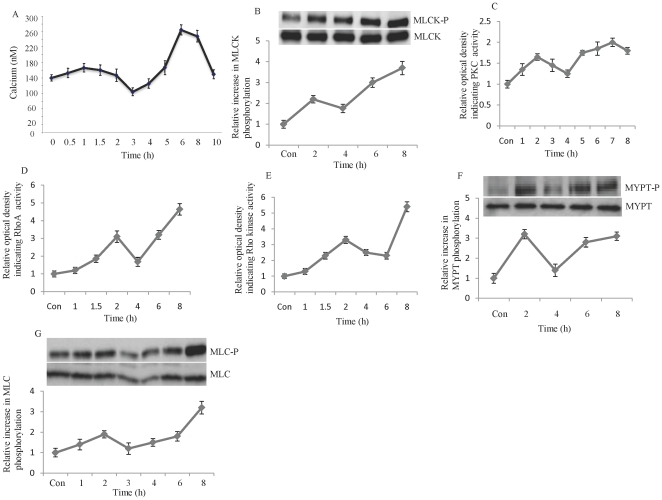
Influenza infection increases intracellular calcium, induces phosphorylation of MLCK, MYPT, and MLC, and activates PKC, RhoA, and Rho kinase. HUVECs were infected with influenza virus (MOI, 10) and were collected at the indicated times points [after infection] for calcium measurement by using fluo-3/Fura. Red fluorescence ratios (A), blotting for MLCK phosphorylation (B), and measurement of activities of PKC (C), RhoA (D) and Rho kinase (E). Influenza infection increases MYPT and MLC phosphorylation. HUVECs were infected with influenza virus (MOI, 10) and after the indicated time were lysed and used for MYPT-P (F) and MLC-P (G) blotting. N = 4 for each experiments.

To facilitate infection, bacterial and viral pathogens induce remodeling of the actin cytoskeleton in the cytoplasm of host cells. The effects of ERK activation on phosphorylation of MLCK, MLC, and cell motility have been demonstrated [6], [7]. We sought to investigate the effects of influenza on signaling pathways involved in actin cytoskeleton function. Infection of HUVECs with influenza virus (multiplicity of infection [MOI], 10) increased the intracellular concentration of calcium, a factor critical for contraction of the cytoskeleton, and led to phosphorylation of MLC kinase (Fig. 3, A and B). Thus, we studied possible alterations in other signaling pathways that are involved in actin cytoskeleton remodeling, particularly MLC phosphorylation. The phosphorylation of MLC kinas was accompanied by activation of PKC, RhoA GTPase, Rho kinase, and the phosphorylation/inactivation of the myosin-phosphatase targeting (MYPT) subunit of MLC phosphatase (Fig. 3, C–F). In addition, MLC phosphorylation was simultaneously induced by influenza virus (Fig. 3, G). The phosphorylation of MLC and the activation of the above-mentioned signaling pathways followed a biphasic pattern that corresponded with early and late stages of the viral replication cycle. Infection of MDCK cells with influenza virus resulted in the phosphorylation of MLC, MLC kinase, and MLC phosphatase (data not shown). These studies indicate that influenza infection leads to phosphorylation of MLC, which correlates with the activation of MLC kinase and the inactivation of MLC phosphatase. In addition to ERK activation [12], influenza infection activates the signaling pathways upstream of MLC phosphorylation-PKC and RhoA/Rho kinase.

### Influenza infection leads to remodeling of the actin cytoskeleton

**Figure 4:**
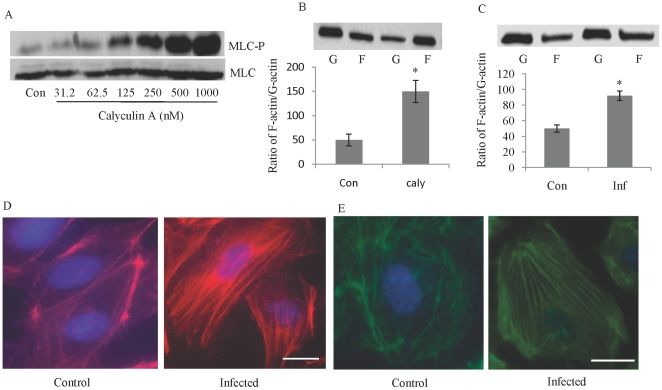
Infection of HUVECs with influenza virus leads to remodeling of actin cytoskeleton. Treatment of HUVECs with calyculin A induces MLC phosphorylation (A). HUVECs were treated with the indicated concentration of calyculin A for 15 minutes. MLC phosphorylation leads to an increase in the F-actin to G-actin ratio (B). HUVECs were treated with 500 nM of calyculin A (caly) or DMSO (Con) and, after 15 minutes, were homogenized in F-actin stabilization buffer and then centrifuged to separate the F-actin [F] from the G-actin [G] pool. The fractions were separated by SDS-PAGE, and actin was quantified by Western blot. Influenza infection increased actin polymerization, the formation of actin stress fibers, and the assembly of myosin. HUVECs were infected with influenza (MOI, 10); 8 h later, the F/G ratio was measured (C). Actin fiber formation (D) and assembly of non-muscle myosin II (E) were assessed by specific staining using fluorescence microscopy. Actin fibers were detected by Texas-red conjugated phalloidin. Primary antibodies were used to bind myosin II, and FITC-conjugated secondardy antibodies were used for detection. Hoechst 33342 was used as nuclear counterstain (blue). Bar, 10 µm. *P<0.01. N = 4 for each experiments.

Viruses use multiple mechanisms of movement within cells, ranging from diffusion within the cytosol to active transport along cytoskeletal filaments. Globular-actin (G-actin) readily polymerizes under physiologic conditions to form filamentous-actin (F-actin) with the concomitant hydrolysis of ATP. To determine the effect of MLC phosphorylation on the actin cytoskeleton, we increased MLC phosphorylation by treating HUVECs with calyculin A (an inhibitor of MLC phosphatase) and then examined G-actin polymerization. Calyculin A treatment of HUVECs resulted in the induction of MLC phosphorylation and G-actin polymerization (Fig. 4, A and B). Infection of HUVECs with influenza virus also led to an increase (almost two fold) in the ratio of F actin to G actin and to the formation of actin stress fibers, as detected by phalloidin staining (Fig. 4, C and D). The interaction of actin and myosin required the polymerization of actin and the assembly of myosin thick filaments. Myosin thick filaments are dynamic cytoskeletal structures that undergo regulated assembly [30], which depends on phosphorylation of MLC [31]. Influenza infection of HUVECs increased the assembly of myosin II, as indicated by immunofluorescence studies (Fig. 4 E). We found that influenza infection of MDCK cells also led to an increase in actin fiber formation and the ratio of F actin to G actin (data not shown). These experiments indicate that influenza infection leads to remodeling of the actin cytoskeleton.

### Engagement of sialic acid leads to phosphorylation of ERK and MLC

**Figure 5:**
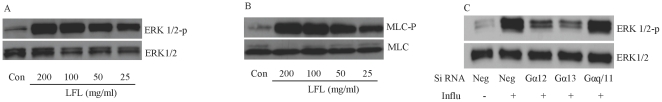
Engagement of sialic acid leads to phosphorylation of ERK and MLC. Treatment of HUVECs with Limax flavus lectin (LFL) leads to ERK and MLC phosphorylation (A, B). HUVECs were treated for 15 min with the indicated concentration of LFL and then lysed. G protein α12/13 mediates influenza-induced ERK phosphorylation. (C) HUVECs were tranfected with the indicated siRNA; 24 h later the cells were infected with influenza virus (MOI, 10) and then collected after 8 h. The pictures are representative of 4 independent experiments.

The viral surface glycoprotein hemagglutinin mediates cellular adhesion by recognizing and binding to sialic residues on the cell surface. The accumulation of influenza hemagglutinin on the surface of infected cells results in the activation of the Raf\MEK\ERK signaling cascade [32]. To determine if MLC phosphorylation is mediated by the interaction of hemagglutinin and sialic acid, we exposed HUVECs to Limax flavus lectin (LFL), which is a specific ligand for sialic acid. Exposure of HUVECs to LFL led to phosphorylation of ERK and MLC in a dose-dependent manner (Fig. 5A, B).

### Heterotrimeric G proteins may play a role in influenza-induced ERK phosphorylation

Signaling via the Ras/Raf/MEK/ERK pathway is commonly initiated by receptor tyrosine kinases or by G-protein–coupled receptors [33]. Heterotrimeric G protein α (Gα) is implicated in Ca^2+^ flux, cytoskeletal functions, and activation of Rho family of GTPases [34]. To understand the role of heterotrimeric G proteins in influenza-induced ERK phosphorylation, we inhibited the expression of Gα 12/13 and Gαq/11 by using siRNA-silencing techniques. Inhibition of Gα 12/13 but not Gαq/11 attenuated influenza-induced ERK phosphorylation in HUVECs (Fig. 5C).

### The inhibition and induction of MLC phosphorylation inhibits and enhances influenza proliferation, respectively

**Figure 6:**
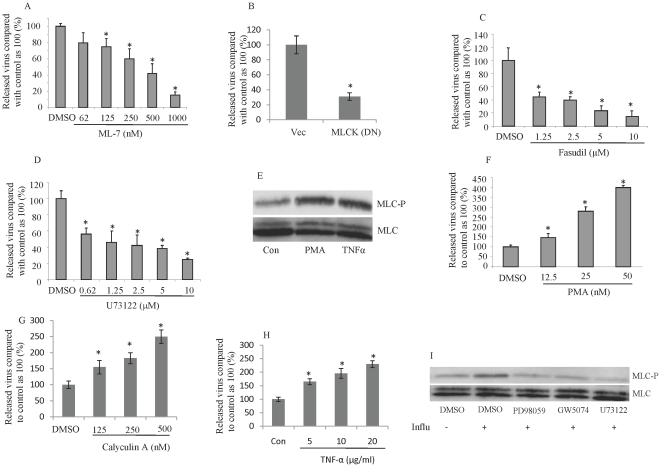
Inhibition or induction of MLC phosphorylation inhibits or enhances influenza proliferation, respectively. Inhibition of MLCK leads to inhibition of influenza replication. MDCK cells were treated with serial dilutions of the MLC kinase inhibitor ML7 for 16 h (A) or were transfected with a DN mutant of MLCK for 24 h (B). The cells were infected with influenza virus (MOI, 0.01), and virus yield was measured 24 h later by RT-PCR. Inhibitors of Rho kinase and phospholipase C inhibitors inhibit influenza proliferation. MDCK cells were treated with the indicated concentrations of Fasudil (C) or the phospholipase C inhibitor, U73122 (D) and 16 h later were infected with influenza virus (MOI, 0.01). Treatment of HUVECs with PMA and TNF-α leads to phosphorylation of MLC. (E) HUVECs treated for 15 min with 5 nM PMA or 20 ng/ml TNF-α. Treatment of HUVECs with PMA, calyculin A or TNF-α enhances influenza proliferation. (F–H) HUVECs were treated with the indicated concentrations of PMA (F), calyculin A (G) or TNF-α (H) for 30 minutes and then infected with influenza (MOI, 0.01). After 24 h, virus yield was measured by RT-PCR. Influenza-induced MLC phosphorylation is inhibited by inhibitors of MEK/ERK, Raf kinase and phospholipase C. (I) HUVECs were pretreated with PD98059(5 Mm, 2 h), GW 5074 or U73112 (10 µM each for 2 h), infected with 10 MOI of influenza virus, and collected 8 h later. *P<0.01; N = 4 for each experiments.

Several anti-influenza compounds have been described, including those that inhibit intracellular calcium, PKC, and Raf/MEK/ERK, as well as calmodulin antagonists and donors of nitric oxide (NO) [12], [14], [15], [16], [17]. As shown in Fig. 1, the common denominator for all of these compounds is their involvement in phosphorylation/dephosphorylation of MLC. Therefore, we tested the hypothesis that inhibiting MLC phosphorylation by blocking signaling pathways that regulate MLC phosphorylation prevents the proliferation of the influenza virus. We found that inhibiting MLC kinase by treating MDCK cells with MLC kinase inhibitor (ML-7) or by expressing DN MLCK prevented influenza proliferation (Fig. 6, A and B). Similar results were obtained when MDCK cells were treated with a Rho kinase (fasudil) or a phospholipase C inhibitor (U73122) (Fig. 6, C and D). To further study this issue, we used real-time polymerase chain reaction to examine the effect of inhibiting other pathways involved in MLC phosphorylation on influenza replication. We found that influenza proliferation was inhibited in a dose-dependent manner by treating MDCK cells with the following: BAPTA-AM (an intracellular calcium chelator) or verapamil (a calcium-channel blocker), C3 exoenzyme (inhibits RhoA); trifluoperazin dimaleate (inhibits calmodulin); bisindolylmaleimide I (inhibits PKC); U0126 and PD98059 (inhibit MEK/ERK); GW 5074 (inhibits Raf-1); or cytochalasin D (inhibits actin polymerization) (table 1, data not shown). In addition, treatment of MDCK cells with an NO donor (sodium nitroprusside) or an activator of PKG (8-Br-cGMP) inhibited influenza proliferation (table 1, data not shown).

**Table 1:** Inhibition of signaling pathways that are involved in MLC phosphorylation or inhibition of actin polymerization led to dose-dependent restriction in influenza virus proliferation.

	Inhibitor/chemical	Signaling cascade/effect	Concentrations	Pretreatment (h)
1	C3 exoenzyme	Rho A GTPase/inhibitor	0–20 µg/ml	2
2	U0126	Mek1/2/inhibitor	0–20 µM	2
3	PD98059	Mek/ERK/inhibitor	0–5 µM	8
4	Bisindolylmaleimide I	Protein kinase C/inhibitor	0–10 µM	2
5	GW 5074	Raf-1/inhibitor	0–10 µM	2
6	Trifluoperazin dimaleate	Calmodulin/antagonist	0–20 µM	8
7	BAPTA-AM	Intracellular calcium/chelator	0–5 µM	0.5
8	Verapamil	Calcium channel/blocker	0–50 µM	8
9	Sodium nitroprusside	Nitric oxide/donor	0–100 µM	2
10	8-Br-cGMP	Protein kinase G/activator	0–100 µM	2
11	Cytochalasin D	Actin polymerization/inhibitor	0–30 µM	0.5

MDCK cells were treated with the solvent used for dissolving the chemicals, DMSO or PBS (control) or serial dilutions of the indicated chemicals for the indicated time. The cells were infected with influenza A virus (MOI, 0.01) and 24 h after the infection, RT-PCR was used to measure the virus yield in the medium. The proliferation of influenza virus was inhibited in a dose-dependent manner for all chemicals (graphs not shown). N = 4 for each experiment.

Next, we tested whether increased MLC phosphorylation also increased proliferation of influenza virus. Thus, we induced MLC phosphorylation by treating MDCK cells with an MLC phosphatase inhibitor (calyculin A), a PKC activator (PMA), or TNF-α. Treatment of MDCK cells with either calyculin A, PMA or TNF-α increased phosphorylation of MLC (Fig. 6 E, data for calyculin A not shown). Furthermore, addition of these agents to MDCK cells dose-dependently increased proliferation of the influenza virus (Fig. 6, F–H). These findings suggest that MLC phosphorylation is critical for influenza proliferation.

It has been well established that inhibition of RhoA GTPase, calcium/calmodulin and PKC, or increase in NO or cGMP inhibit stimuli-induced MLC phosphorylation [35], [36], [37]. However, for the less-studied inhibitory compounds that we used in the above-mentioned experiments, we studied their effects on MLC phosphorylation after influenza infection. Pretreatment of HUVECs with Raf-1 inhibitor, GW 5074, phospholipase C inhibitor, U73122 and MEK/ERK inhibitor, PD98059 attenuated influenza-induced MLC phosphorylation (Fig. 6 I). These experiments indicate that the compounds that inhibited influenza proliferation have the ability to inhibit MLC phosphorylation. Induction or inhibition of MLC phosphorylation with all the above-mentioned agents in HUVECs yielded similar results (data not shown).

### Influenza-induced MLC phosphorylation is mediated by HRas, RhoA, and PKC-activation

**Figure 7:**
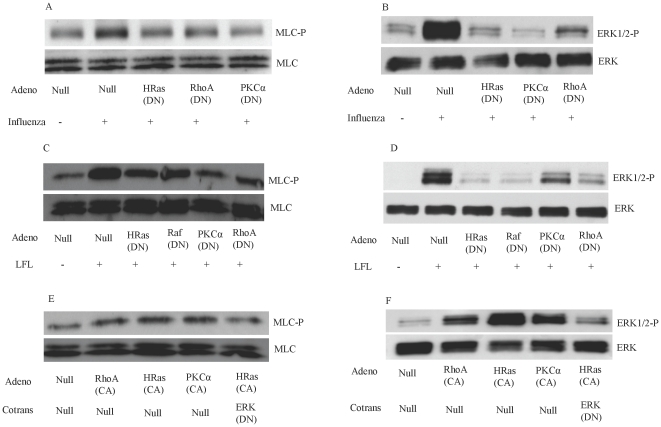
HRas activation increases phosphorylation of MLC. Influenza-induced MLC phosphorylation is inhibited by inhibiting HRas, RhoA, and PKC. (A, B) HUVECs were transduced with the indicated adenoviruses and infected 48 h later with influenza virus (MOI 10). The cells were collected 8 h later for blotting. Limax flavus lectin (LFL)–induced MLC and ERK phosphorylation depends on HRas, Raf-1, PKC-α, and RhoA. (C, D) HUVECs were transduced with the indicated adenoviruses; 48 h later, the cells were treated for 15 min with LFL [100 mg/ml]. Activation of HRas, RhoA, and PKC-α leads to MLC and ERK phosphorylation. (E, F) Western blots performed 48 hours after HUVECs were transduced with empty adenovirus vector (Null) or the indicated adenoviruses. *P<0.01; N = 4 for each experiment.

To investigate the signaling mechanism underlying the phosphorylation of MLC after influenza infection, we sought to examine the signaling pathways upstream of MLC phosphorylation. Previous studies demonstrated that activation of Raf/MEK/ERK is crucial for proliferation of the influenza virus [12], [38], [39]. However, it is believed that ERK activation is not due to the accumulation of viral RNA [32]. The Raf/MEK/ERK signal cascade can be activated by Ras or PKC-α [40], [41], the latter of which has been reported to be the upstream signal that leads to Raf/MEK/ERK activation after influenza infection [32]. On the other hand, it has been reported that RhoA activation leads to sequential activation of phospholipase C-ε, PKC-α, and ERK [42]. RhoA activation also promots ERK phosphorylation by activation of MEKK1 [43], [44]. To determine the roles of HRas, PKC-α, and RhoA in influenza-induced ERK and MLC phosphorylation, DN mutants of these genes were overexpressed in HUVECs. We found that influenza-induced ERK and MLC phosphorylation was attenuated when HRas, PKC-α, or RhoA were inhibited by their DN mutants (Fig. 7 A, B). These findings suggest that influenza-induced MLC phosphorylation is mediated by HRas, PKC-α, or RhoA signal transduction pathways.

The increase in ERK phosphorylation during the late phase of the influenza replication cycle has been shown to depend on accumulation of hemagglutinin on the cell surface of infected cells [32]. Therefore, it may be argued that the reduction in influenza-induced ERK phosphorylation in the above-mentioned experiment is due to the inhibitory effects of these pathways in the proliferation of influenza virus (via other mechanisms) and not due to their direct effect in influenza-induced ERK phosphorylation. Thus, we simulated influenza-induced ERK and MLC phosphorylation by using LFL to bind sialic acid. LFL-induced phosphorylation of MLC and ERK was attenuated when HUVECs were transduced with the DN mutants of HRas, Raf-1, PKC-α, and RhoA (Fig. 7 C, D). The roles of RhoA and PKC activation in MLC phosphorylation are well documented [4]. However, the role of HRas\Raf\MEK\ERK activation in MLC phosphorylation has not been fully studied. Thus, we overexpressed the CA mutant of HRas, RhoA, and PKC-α in HUVECs to determine their effect on ERK and MLC phosphorylation. The activation of HRas, RhoA, and PKC-α led to phosphorylation of ERK and MLC (Fig. 7 E, F). HRas-induced phosphorylation of MLC was attenuated when ERK phosphorylation was inhibited with DN ERK (Fig. 7 E), indicating a critical role for ERK in HRas-induced MLC phosphorylation. These experiments suggest that HRas\Raf\MEK\ERK, PKC-α, and RhoA are critical for influenza-induced ERK and MLC phosphorylation.

### MLC phosphorylation is critical for nuclear export of influenza ribonucleoprotein

**Figure 8:**
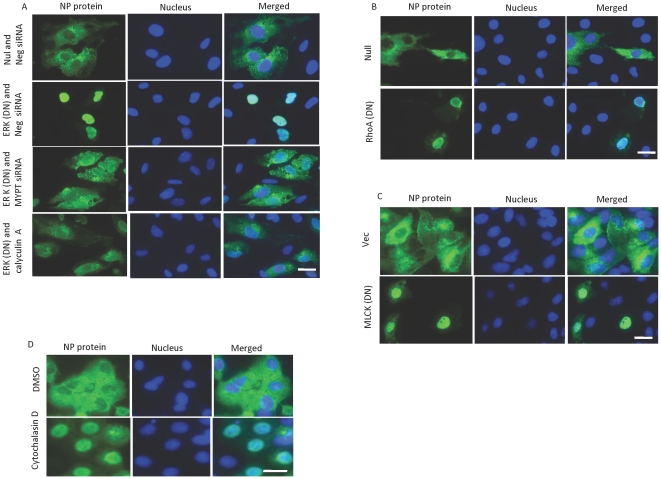
Inhibition of ERK and MLC phosphorylation and actin polymerization leads to nuclear retention of influenza RNP complexes. The effect of ERK on the nuclear export of influenza RNP complexes is mediated by MLC phosphorylation. (A) HUVECs cells were cultured on chamber slides, transduced with the indicated adenovirus, and, 24 laters, transfected with the indicated siRNA. After 24 h, the cells were infected with influenza virus (MOI 10). Calyculin A (0.5 nM) was added to the indicated sample 30 minutes before the infecton. Eight h later, the cells were stained for the presence of influenza nucleoprotein (NP) protein (green). Inhibition of RhoA leads to inhibition of the nuclear export of influenza RNP complexes. (B, C) HUVECs were transduced with the indicated adenoviruses (B) and constructs (C) and were processed as described above. Polymerized actin is necessary for the nuclear export of influenza RNP complexes. (D) MDCK cells were infected with influenza virus (MOI 10) and treated 2 h later with DMSO or 30 µM of cytochalasin D. After 6 h, the cells were fixed and stained. Hoechst 33342 was used as nuclear counterstain (blue). Bar, 5 µm; N = 4 for each experiment.

Replication and transcription of the influenza virus genome occurs exclusively within the nucleus of the infected cells. The viral RNA genome, polymerase subunits, and nucleoprotein form ribonucleoprotein (RNP) complexes, which are then exported from the nucleus to form budding virions at the cell membrane. This process requires viral activation of the cellular Raf/MEK/ERK signaling cascade. Accordingly, blocking this signaling pathway inhibits the export of RNP complexes and reduces titers of progeny virus [12]. To determine which part of the viral replication cycle requires MLC phosphorylation, we examined the intracellular trafficking of the influenza virus in HUVECs. Immunofluorescence studies showed that inhibiting ERK with DN ERK blocked the nuclear export of influenza RNP complexes (Fig. 8 A, two top rows). Similarly, inhibiting MLC phosphorylation by blocking RhoA or MLC kinase restricted the nuclear translocation of RNP complexes (Fig. 8 B, C).

The contractile function of the actin cytoskeleton depends on MLC phosphorylation and the presence of actin filaments. Impeding actin polymerization inhibits the internalization of influenza virus [18]. To determine the role of the actin cytoskeleton in the nuclear export of influenza RNP complexes, we inhibited actin polymerization with the use of cytochalasin D. Treatment of MDCK cells with cytochalasin D 2 hours after infection with influenza delayed the nuclear translocation of RNP complexes (Fig. 8 D), indicating the importance of the actin cytoskeleton in this process. In MDCK cells, nuclear export of influenza RNP complexes was also inhibited by blocking Rho A and MLC kinase (data not shown).

### MLC phosphorylation mediates the role of HRas/Raf/MEK/ERK in influenza proliferation

**Figure 9:**
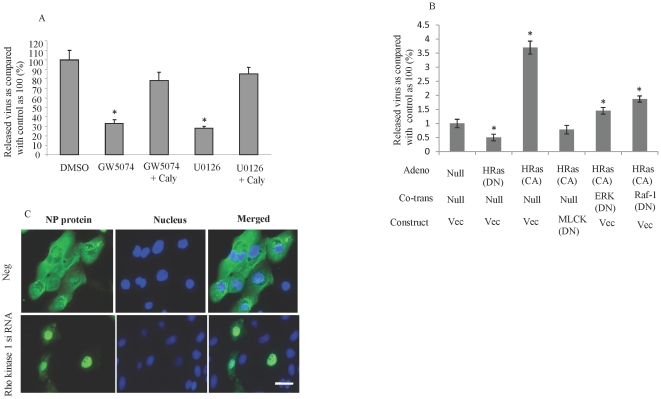
Induction of MLC phosphorylation reverses the inhibitory effects of HRas\ERK on influenza proliferation. The anti-influenza effects of Raf kinase and MEK/ERK inhibitors were reversed when HUVECs were treated with an MLC phosphatase inhibitor. (A) MDCK cells were treated as indicated GW5074 and U0126 at 10 µM each overnight; calyculin A at 0.5 µM for 1 h] and then infected with influenza (MOI, 0.01). After 24 h, virus yield was measured in the medium by using RT-PCR. HRas-enhanced influenza proliferation is attenuated by inhibition of MLC kinase, ERK and Raf-1. (B) MDCK cells were seeded on 24-well plates, transduced with the indicated adenovirus, and transfected 24 h later with the indicated constructs. The cells were infected with influenza A virus (MOI, 0.01) and 24 h after the infection, RT-PCR was used to measure the virus yield in the medium. Inhibition of Rho kinase 1 in human bronchial epithelial cells leads to inhibition of the nuclear translocation of influenza RNP complexes. (C) Human bronchial epithelial cells were seeded on chamber slides, transfected with the indicated siRNA, and infected 24 h later with influenza virus (MOI, 10). After 8 h, the cells were stained for influenza nucleoprotein (NP) protein. Hoechst 33342 was used as a nuclear counterstain (blue). Bar, 5 µm; *P<0.01; N = 4 for each experiment.

We sought to determine if MLC phosphorylation mediates the role of HRas\Raf\MEK\ERK in influenza proliferation. The reduction in influenza proliferation seen when MDCK cells were treated with the MEK/ERK inhibitor, U0126, or with the Raf kinase inhibitor, GW5074, was reversed when MDCK cells were also treated with the MLC phosphatase inhibitor, calyculin A (Fig. 9 A). Induction of ERK phosphorylation by overexpression of the CA-HRas enhanced influenza proliferation (Fig. 9 B). This effect was reversed when MDCK cells were cotransduced with DN mutants of MLC kinase, ERK, or Raf-1 (Fig. 9 B), suggesting that the effect of HRas activation on influenza virus proliferation is mediated by MLC phosphorylation. In addition, an increase in MLC phosphorylation by treatment of HUVECs with calyculin A or siRNA against MLC phosphatase reversed the inhibitory effect of DN ERK on the nuclear translocation of influenza RNP complexes (Fig. 8 A).

To assess the possible clinical applications of our findings in HUVECs and MDCK cells, we conducted experiments using the NHBE cells because lung epithelial cells are the primary target cell of influenza. In NHBE cells, knocking down Rho kinase with siRNA inhibited the nuclear translocation of influenza RNP complexes (Fig. 9 C), as observed in HUVECs and MDCK cells. Furthermore, influenza infection activated the same signal transduction pathways in NHBE cells as in HUVECs and MDCK cells (data not shown).

## Discussion

In the present study, we have shown that influenza infection activates the signaling pathways that converge to induce MLC phosphorylation and actin cytoskeleton remodeling. Inhibiting MLC phosphorylation appears to be the underlying mechanism of action for a significant group of anti-influenza agents. In particular, we showed that MEK/ERK inhibitors suppressed the proliferation of influenza virus by blocking MLC phosphorylation. In addition, inhibition of MLC phosphorylation led to the nuclear retention of viral RNP complexes in late stages of the viral replication cycle, thus reducing proliferation of the influenza virus.

To our knowledge, we are the first to report the influenza-induced activation of RhoA/Rho kinase and MLC phosphorylation. In addition, we observed that RhoA activation leads to ERK phosphorylation. RhoA had been shown to prevent apoptosis in zebrafish by activating ERK cascade [45]. On the other hand, RhoA has been found to bind and activate the kinase activity of MEKK1 in HEK 293 cells [44]. Scaffold protein MEKK1 is known to phosphorylate MEK1 via its kinase activity and to recruit Ras, Raf1 and MEK1 leading to the promotion of ERK activation [43], [44]. The accumulation of influenza hemagglutinin in the late stages of the viral replication cycle has been shown to lead to phosphorylation of ERK [32]. Using a different approach, we showed that sialic acid, the cell receptor of influenza hemagglutinin, bound to a specific lectin reproduces influenza-induced phosphorylation of ERK and MLC. In support of our findings, previous studies have shown that the interaction of influenza hemagglutinin with sialic acid initiates activation of PKC [10]. Although lectins have a defined specificity for their carbohydrate substrate and are thus deemed highly specific, lectin binding can also be considered nonspecific because numerous proteins may be glycosylated in an identical fashion. Therefore, although chemical specificity is integral to lectin chemistry, the biology is not as restricted as that seen in antibody reactions [46]. Therefore, the results should be interpreted with careful consideration. Our finding that HRas plays a critical role upstream of RAF/MEK/ERK differs from the results of the study by Marjuki and colleagues who reported a prominent role for PKC-α [32]. To study influenza replication in MDCK cells, they used DN mutants of PKC-α and Ras to inhibit their functions. After 6 hours, a greater reduction in viral titer was reported with DN PKC-α than with DN RAS, although the latter was still associated with a 60% reduction in viral titer. For DN Ras this effect did not remain significant after 8 hours. However, in the present study, we showed that activation of HRas, PKC-α, and RhoA are required for influenza-induced ERK phosphorylation. This discrepancy in results may be explained by the methodology used to inhibit HRas; Marjuki and colleagues used a plasmid vector to inhibit HRas, whereas we used an adenovirus, which more efficiently inhibits HRas. The molecular pathways that link hemagglutinin/sialic acid interaction to activation of HRas\Raf\MEK\ERK, PKC-α, RhoA, and Rho kinase pathways are not known. Our studies indicate that inhibition of HRas\Raf\MEK\ERK, PKC-α, or RhoA is sufficient to suppress influenza-induced MLC phosphorylation. In addition, inhibition of MLC phosphorylation by inhibiting any of these pathways leads to restriction in influenza virus replication. This finding suggests that activation of each of these pathways is critical for influenza virus proliferation. Further studies are needed to reveal the molecular mechanisms underlying this phenomenon. We speculate that simultaneous activaton of MLC kinase and inactivation of MLC phosphatase is critical for influenza-induced MLC phosphorylation.Therefore, inhibition of MLC kinase or activation of MLC phosphatase by inhibition of any of HRas\Raf\MEK\ERK, PKC-α and RhoA signal transductions leads to suppression of influenza-induced MLC phosphorylation. The other possible scenario is that influenza virus uses the crossroads between these pathways to activate HRas\Raf\MEK\ERK, PKC-α and RhoA signal transductions simultaneously. Therefore, inhibition of any of these pathways leads to inhibition of the others and attenuation of influenza-induced MLC phosphorylation. Because of how these pathways intersect, several molecular models can be proposed for their activation by influenza. For example, our results suggest that of the heterotrimeric G proteins, Gα12/13 is critical for influenza-induced ERK activation. Activaton of Gα12/13 leads to RhoA and phospholipase C-ε activation [34], [47]. Phospholipase C-ε is known to activate Ras and PKC [48], [49]. Besides, some activation mechanisms of RhoA by Ras had been reported [50], [51].

We found that MLC phosphorylation is critical for the export of influenza RNP complexes from the nucleus. However, it is not clear how MLC phosphorylation contributes to the nuclear translocation of influenza RNP complexes. Many bacterial and viral pathogens facilitate infection by inducing actin polymerization in the cytoplasm of host cells. The actin cytoskeleton plays a critical role in endocytosis and in intracellular trafficking of viruses [52], [53]. Actin is a highly conserved protein and is one of the main components of the cytoplasm and nucleus in eukaryotic cells. In the nucleus, actin is involved in several processes, including transcription and transcription regulation, RNA processing and export, intranuclear movement, and maintenance of nuclear structure [54], [55]. Early studies indicated that actin or actin-like structures were involved in intranuclear transport and nuclear export of viruses [56], [57]. It has been suggested that viral capsids use myosin-based–directed transport to travel along nuclear actin filaments [58]. In addition, increasing evidence suggests that the cytoplasmic and nuclear pools of actin are functionally linked. The signaling pathways for both pools are similar. The nucleus contains several signaling molecules, including phosphoinositides, Ca^2+^, and small GTPases, all of which have well-characterized effects on actin dynamics in the cytoplasm [59]. Therefore, it is tempting to speculate that phosphorylation of MLC and actin remodeling in the cytoplasm leads to corresponding changes in the nucleus, facilitating the nuclear transport of influenza either by alteration in gene expression or through an actin-dependent intranuclear transport system.

In conclusion, our studies indicate that phosphorylation of MLC is critical for the proliferation of influenza virus, and inhibition of MLC phosphorylation is the mechanism underlying the effects of many anti-influenza agents.
